# Institutional Notes and News

**Published:** 1910-02-05

**Authors:** 


### Institutional Notes and News.

**Edinburgh Eye and Ear Infirmary.**

The annual report of the Edinburgh Eye, Ear, and Throat Infirmary was adopted at the yearly meeting of contributors which was held on January 24. Patients to the number of 4,771 had attended the Infirmary, while almost twice that number of visits was recorded. The new wards had proved a great success in the two years since their acquisition, and the operation theatre of similar date had been a great improvement, and a large number of major operations in it had been performed last year. The ordinary revenue was £398, an increase of £94 on their previous income, and the ordinary expenditure was £407, or £14 less than in 1908. Each patient, therefore, showed an average cost of only 1s. 8½d., and it is interesting to note that the patients themselves have given of their own accord £75 towards the expenses. The very satisfactory financial statement was principally due to a donation of £100 from Mr. J. C. Wordie, who gave a similar sum towards the reduction of the Infirmary's debt. The bond over the Infirmary had been brought down to £250 by a grant of £500 from the trustees of Mr. J. Dick, of Glasgow. The ordinary income, nevertheless, is still in arrear of the expenditure, but it is hoped the economy of the institution as well as the character of its work will appeal to the generosity of the public.

**The Need for Extension at Coventry.**

The pressing need for completing the scheme of extension at the Coventry and Warwickshire Hospital, owing to its overcrowded condition, was seriously discussed on January 12 at the General Committee Meeting. Mr. A. Herbert, again elected Chairman of the hospital, said :—"I wish members to realise that not only are we quite unable to cope with the work, but the patients in the hospital are crowded in a manner quite insanitary and totally contrary to what ought to be the case in the light of modern knowledge. We are not making any progress with the extension fund. Something must be done. You must not lose sight of the fact that there is likely to be a large influx of labour into the city in the near future." The house committee reported that for the week ending on December 28, while the total number of available beds was 85, the daily average number of patients was 83.1. It was decided to consider the possibility of arranging a demonstration in aid of the funds, and the last Sunday in April was chosen for the yearly collection.

**Sussex Throat and Ear Hospital.**

The Brighton, Hove, and Sussex Throat and Ear Hospital, at its recent annual meeting, was able to announce that the balance due to the bankers on the building fund had been reduced to £615, with the help of a legacy of £135 left by the late Miss H. Dennett. Notwithstanding, the financial year begins with a debt of £58, which makes the balance of £91 with which last year began an extremely lucky one. Of the total receipts, £979, the in-patients supplied £88, and the out-patients, including the contents of boxes placed in the building, £78. It may be remarked at this point that the number of in-patients last year was 343. The operation theatre has been ventilated, and the worn-out baths attached to the wards have been taken away and replaced by new ones of up-to-date quality. The report points out that increased support is needed, and should be forthcoming as soon as the public has been taught to realise that the Throat and Ear Hospital is not only a Brighton institution, but a county one. Patients are too much in the habit of coming to Brighton for subscribers' letters, when they ought by reiterated appeals to make each village realise the duty of its richer members to provide such letters for local use. The Committee hopes soon to take in hand the completion of the buildings—the plans for which have been ready for some years. The Ladies' Linen League, which has supplied the new linen for the hospital, received a sincere vote of thanks for its work. An announcement was also made that £25 had been given in support of the "Lewis Carrol" cot.

**German Hospital, Dalston.**

Baron Bruno von Schröder presided on Tuesday, February 1, at the annual general meeting of this institution, held at Winchester House, E.C. The Secretary read the Committee's report, which stated that the work of the hospital had been maintained at full pressure throughout the year; 1,690 in-patients had been admitted, of whom 498 were English; and 26,108 out-patients had been attended to at the hospital and dispensary. The income amounted to £12,502 7s. 4d., and the expenditure to £12,584 5s. 5d. The Chairman, in moving the adoption of the report, observed that the figures gave undeniable proof of the increasing usefulness of the hospital, and showed the great benefit it bestowed upon the poor suffering Germans, English, and other nationalities. The hospital had now a position in London second to none, and it was acknowledged by everyone that the work was done efficiently, and that the nursing was of a superior character. He trusted that reputation would always be maintained. The Convalescent Home at Hitchin had also proved of the greatest benefit to the patients from the hospital. Although the hospital was an excellent one for curing patients, none the less when a patient arrived at a certain stage of convalescence he needed fresh air, and that he could hardly get at Dalston. In such cases the home at Hitchin supplied a long-felt want. The Chairman added that he was sure that all would join in the Committee's expression of regret at the retirement of Baron von Schröder, as Treasurer and Trustee, and of Mr. A. J. Allen, as Trustee. Baron von Schröder had been connected with the hospital for upwards of fifty years, and had given invaluable aid to the institution on every possible occasion. Mr. Allen had also been an active member of the Committee since 1862. The Chairman also referred to the Committee's decision to proceed with the much-needed extension to the hospital, comprising additional accommodation for patients, nurses, and servants, and rooms for bacteriological, pathological and other work. That had been made possible by the generous and ready response to a special appeal made privately amongst the friends of the hospital. The present total of that fund was £27,847 15s., but further help was needed to meet the increased cost of maintenance which the extension would involve. The report was unanimously agreed to. Complimentary votes to the medical officers, Committee, and others actively connected with the welfare of the institution were passed, and the meeting terminated with an acknowledgment of the Baron's services in the chair.

